# Vasopressor‐Associated Limb Ischemia Resulting in Quadruple Amputation in Suspected Septic Shock

**DOI:** 10.1002/ccr3.73412

**Published:** 2026-09-04

**Authors:** Muni Rubens, Venkataraghavan Ramamoorthy, Krimal Shah, Ashley Rodriguez, Brian Murillo, Karelis Lopez Muniz, Anshul Saxena, Peter McGranaghan, Tina Sanjar

**Affiliations:** ^1^ Herbert Wertheim College of Medicine Florida International University Miami Florida USA; ^2^ Baptist Health South Florida Miami Florida USA; ^3^ Semmelweis Doctoral College Semmelweis University Budapest Hungary

**Keywords:** hemophagocytic lymphohistiocytosis, obesity paradox, quadruple amputation, vasopressor‐associated limb ischemia

## Abstract

Vasopressor‐associated limb ischemia (VALI) is a rare but devastating complication of vasopressor therapy in septic shock. This case shows catastrophic four‐limb amputation in a 26‐year‐old obese male with suspected septic shock. This case illustrates the complex interplay between prolonged high‐dose vasopressor therapy, morbid obesity, and cytokine‐mediated microvascular injury in VALI.

## Key Clinical Message

Morbid obesity and hyperinflammatory states may markedly increase the risk of vasopressor‐associated limb ischemia during prolonged high‐dose vasopressor therapy in septic shock, underscoring the importance of individualized dosing, watchful perfusion monitoring, and early recognition to prevent catastrophic ischemic complications such as four‐limb amputation.

## 1 Introduction

Vasopressors are essential medications in the management of septic shock, with norepinephrine recommended as the first‐line agent [1]. While these medications restore hemodynamic stability and maintain organ perfusion, they carry the risk of serious complications, including limb ischemia and necrosis [2]. Vasopressor‐induced peripheral ischemia represents a rare but devastating complication, with a reported incidence of approximately 0.25% among patients receiving vasopressor therapy [3]. However, among those who develop this complication, the consequences are severe: up to 70% of survivors require amputation, and mortality rates remain exceedingly high [4].

The pathophysiology involves both direct vasoconstrictive effects through α‐adrenergic receptor activation and underlying patient factors [5]. Excessive peripheral vasoconstriction, particularly in acral regions where microvascular perfusion is already compromised by shock, can lead to symmetrical peripheral gangrene characterized by symmetrical distal ischemic damage without large vessel occlusion [4]. Risk factors include higher peak doses and prolonged duration of vasopressor therapy, particularly with norepinephrine, epinephrine, and dopamine [3, 4]. Notably, baseline demographics and medical comorbidities do not differ significantly between patients who develop limb necrosis and those who do not, suggesting that medication‐specific factors play a predominant role [3].

We present a case of catastrophic four‐limb amputation following vasopressor therapy in a patient with suspected septic shock, highlighting the clinical challenges and devastating outcomes associated with this rare complication.

## 2 Case History/Examination

A 26‐year‐old morbidly obese male (BMI 40.82 kg/m^2^) presented with a 3‐day history of fever, chills, myalgia, headache, palpitations, and shortness of breath, initially evaluated at an urgent care center before clinical deterioration prompted hospital admission. On presentation, the patient was critically ill with hypotension (BP 80/45 mmHg), tachycardia (HR 143 bpm), tachypnea (RR 23 breaths/min), and hyperthermia (39.4°C), requiring supplemental oxygen. Physical examination revealed severe distress, a rash in the right axillary region, and posterior pharyngeal erythema, raising concern for a systemic infectious process with possible toxic shock physiology.

Given the patient's hemodynamic instability and multiorgan involvement, broad‐spectrum antimicrobial therapy and aggressive supportive management were initiated. Despite these measures, the patient remained critically ill, prompting extensive diagnostic evaluation. Computed tomography imaging of the chest demonstrated bilateral linear atelectasis without focal infiltrates but revealed inflammatory changes in the right axillary region suggestive of cellulitis. Abdominal and pelvic CT imaging did not demonstrate an intra‐abdominal source of infection, while neck CT showed mucosal thickening consistent with pharyngitis. Transthoracic echocardiography revealed a preserved ejection fraction of 50%–55% without regional wall motion abnormalities. Abdominal ultrasonography identified splenomegaly, raising suspicion for an underlying hyperinflammatory or hematologic process.

Laboratory evaluation was notable for profound leukocytosis (57.11 × 10^3^/μL) with neutrophilic predominance and bandemia, severe anemia (hemoglobin 8.5 g/dL), and marked thrombocytopenia (17,000/mm^3^), indicating cytopenias across multiple cell lines. Biochemical studies revealed acute kidney injury (creatinine 2.92 mg/dL), metabolic acidosis with elevated lactate (7.4 mmol/L), and significant hepatic dysfunction (AST 238 U/L, ALT 129 U/L, alkaline phosphatase 321 U/L, total bilirubin 4.2 mg/dL). Coagulation abnormalities were present with prolonged PT (22.7 s), elevated D‐dimer (3.47 FEU), and hypofibrinogenemia (124 mg/dL), consistent with consumptive coagulopathy. Inflammatory markers were markedly elevated, including procalcitonin (60.8 ng/mL), ferritin (1736 ng/mL), triglycerides (580 mg/dL), LDH (1315 U/L), and CRP > 200 mg/dL, indicating a severe systemic inflammatory response. Arterial blood gas analysis demonstrated severe acidemia (pH 7.07) with metabolic acidosis and hypoxemia.

Given the constellation of persistent fever, bicytopenia, hyperferritinemia, hypertriglyceridemia, hypofibrinogenemia, and splenomegaly, a diagnosis of hemophagocytic lymphohistiocytosis (HLH) was strongly suspected. The patient met at least five of the eight HLH‐2004 diagnostic criteria, including fever ≥ 38.5°C, cytopenias affecting ≥ 2 cell lines, elevated ferritin, elevated triglycerides and/or low fibrinogen, elevated soluble IL‐2 receptor levels (> 20,000 U/mL), and splenomegaly. However, these findings substantially overlap with the hyperinflammatory phenotype of severe sepsis complicated by disseminated intravascular coagulation and ischemic hepatitis (“shock liver”). In the overall clinical context, these abnormalities were considered more likely to reflect a profound cytokine storm induced by polymicrobial sepsis.

Microbiologic evaluation revealed negative blood and urine cultures; however, sputum cultures grew methicillin‐sensitive *Staphylococcus aureus* . Additionally, plasma microbial cell‐free DNA testing (Karius test) identified polymicrobial organisms, including *Escherichia coli* , *Bacteroides thetaiotaomicron* , *Bacteroides vulgatus* , and *Haemophilus parainfluenzae* , suggesting a possible occult polymicrobial infectious source contributing to the hyperinflammatory state.

Based on clinical, laboratory, and microbiologic findings, the patient was diagnosed with severe septic shock complicated by morbid obesity contributing to a hyperinflammatory state. The case highlights the importance of early recognition of hyperinflammatory state in critically ill patients with sepsis, particularly when there is evidence of cytopenias, hyperferritinemia, and multiorgan dysfunction. Prompt identification and initiation of appropriate antimicrobial therapy is essential to improving outcomes in this life‐threatening condition.

Written informed consent was obtained from the patient's legally authorized representative for publication of this case report and any accompanying images or clinical information.

## 3 Differential Diagnosis

At presentation, the patient exhibited high‐grade fever, hemodynamic instability, altered systemic perfusion, and multiorgan dysfunction, prompting consideration of several life‐threatening diagnoses. Septic shock was initially suspected and served as the primary working diagnosis given the presence of hypotension, elevated lactate, marked leukocytosis, and significantly elevated procalcitonin, consistent with severe systemic infection. Concurrently, streptococcal toxic shock syndrome and fulminant bacterial pharyngitis with sepsis were strongly considered in light of the patient's fever, rash, and pharyngeal erythema, although no definitive streptococcal source was microbiologically confirmed.

Given the presence of profound cytopenias, hyperferritinemia, hypertriglyceridemia, hypofibrinogenemia, and splenomegaly, secondary infection‐associated hemophagocytic lymphohistiocytosis (IA‐HLH) was considered. Although this diagnosis was supported by HLH‐2004 criteria, the lack of extremely high ferritin levels and improvement of the patient's clinical status was against a diagnosis of IA‐HLH. Thrombotic microangiopathy was also considered due to anemia, thrombocytopenia, and organ dysfunction; however, the laboratory profile and clinical context were more consistent with sepsis‐related coagulopathy rather than a primary microangiopathic process.

Necrotizing soft tissue infection was evaluated given the presence of right axillary inflammatory changes and systemic toxicity but was not definitively supported by imaging or clinical progression. Additionally, autoimmune or rheumatologic disease with macrophage activation syndrome (MAS) was considered in the differential; however, the overall presentation favored severe septic shock with an unknown infectious source.

## 4 Conclusion and Results (Outcome and Follow‐Up)

The patient was managed in the intensive care unit with aggressive resuscitative and supportive measures for severe septic shock complicated by multiorgan failure. On hospital day 1, he developed acute hypoxic respiratory failure requiring endotracheal intubation and initiation of mechanical ventilation. Hemodynamic instability necessitated the use of multiple vasopressors for 12 days, including norepinephrine, phenylephrine, and vasopressin, to maintain adequate mean arterial pressure. Table 1 shows vasopressors, doses, and norepinephrine equivalent dose by hospital day. Given the severity of metabolic acidosis and acute kidney injury, continuous renal replacement therapy (CRRT) was initiated early in the course.

**TABLE 1:** Vasopressors, doses, and norepinephrine equivalent dose by hospital day.

Vasopressor drugs	Day 1	Day 2	Day 3	Day 4	Day 5	Day 6	Day 7	Day 8	Day 9	Day 10	Day 11	Day 12
Norepinephrine (μg/min)	2	2	2	2	2	2	2	2	2	2	2	2
Phenylephrine (μg/min)	20	20	20	20	20	—	—	—	—	—	—	—
Vasopressin (units/min)	—	0.03	0.03	0.03	0.03	0.03	—	—	—	—	—	—
Norepinephrine equivalent dose (μg/kg/min)	0.032	0.107	0.107	0.107	0.107	0.091	0.016	0.016	0.016	0.016	0.016	0.016

*Note:* Norepinephrine equivalent dose = [Norepinephrine + (Phenylephrine/10)/weight] + (Vasopressin × 2.5).

Empiric broad‐spectrum antimicrobial therapy was initiated with piperacillin/tazobactam, vancomycin, and clindamycin to provide coverage against gram‐positive, gram‐negative, and toxin‐producing organisms. Due to persistent clinical instability and concern for resistant or polymicrobial infection, antimicrobial therapy was subsequently escalated to meropenem, after which the patient demonstrated gradual clinical improvement.

**FIGURE 1:**
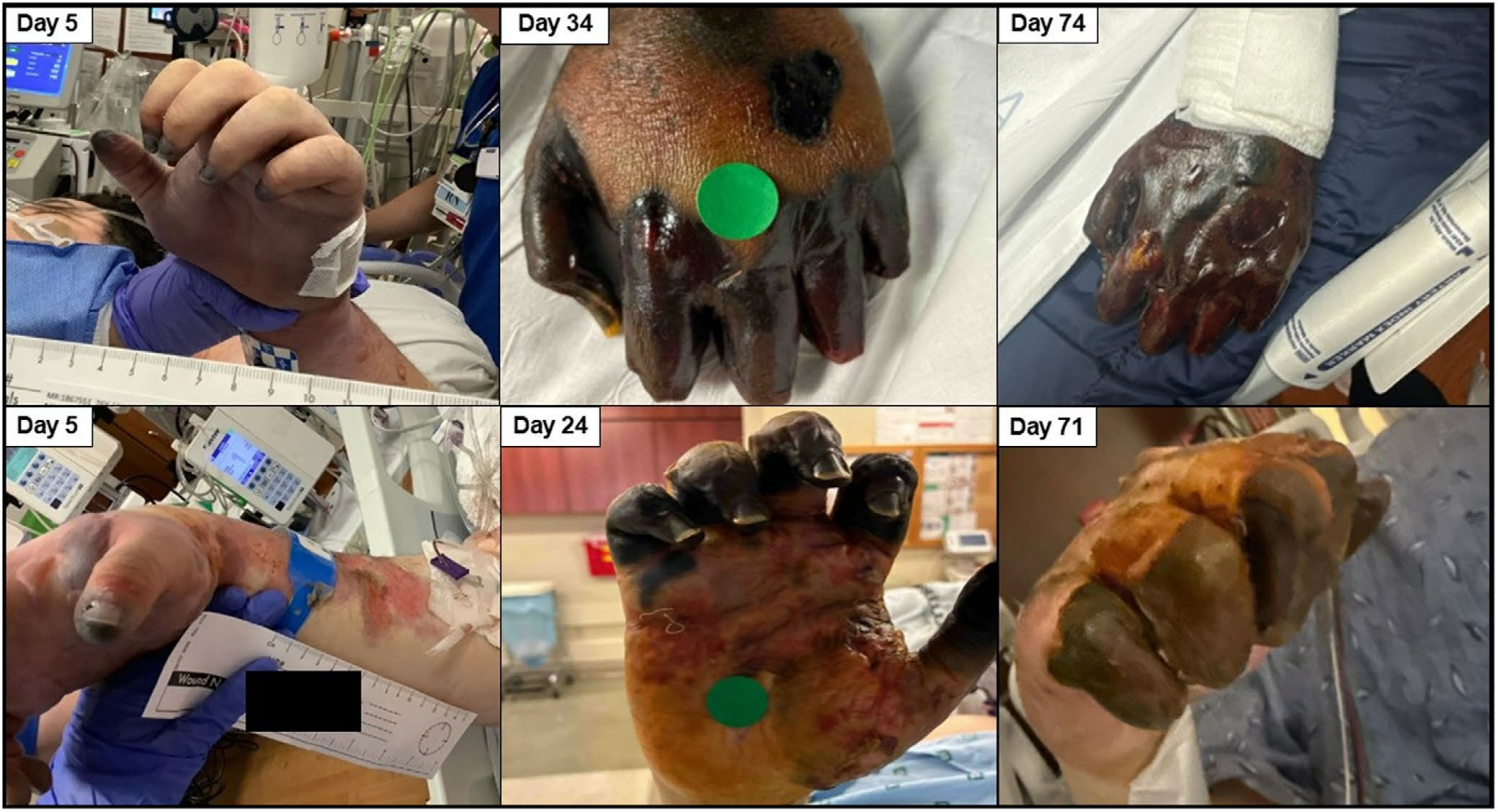
Progression of upper limb ischemia by hospital days.

**FIGURE 2:**
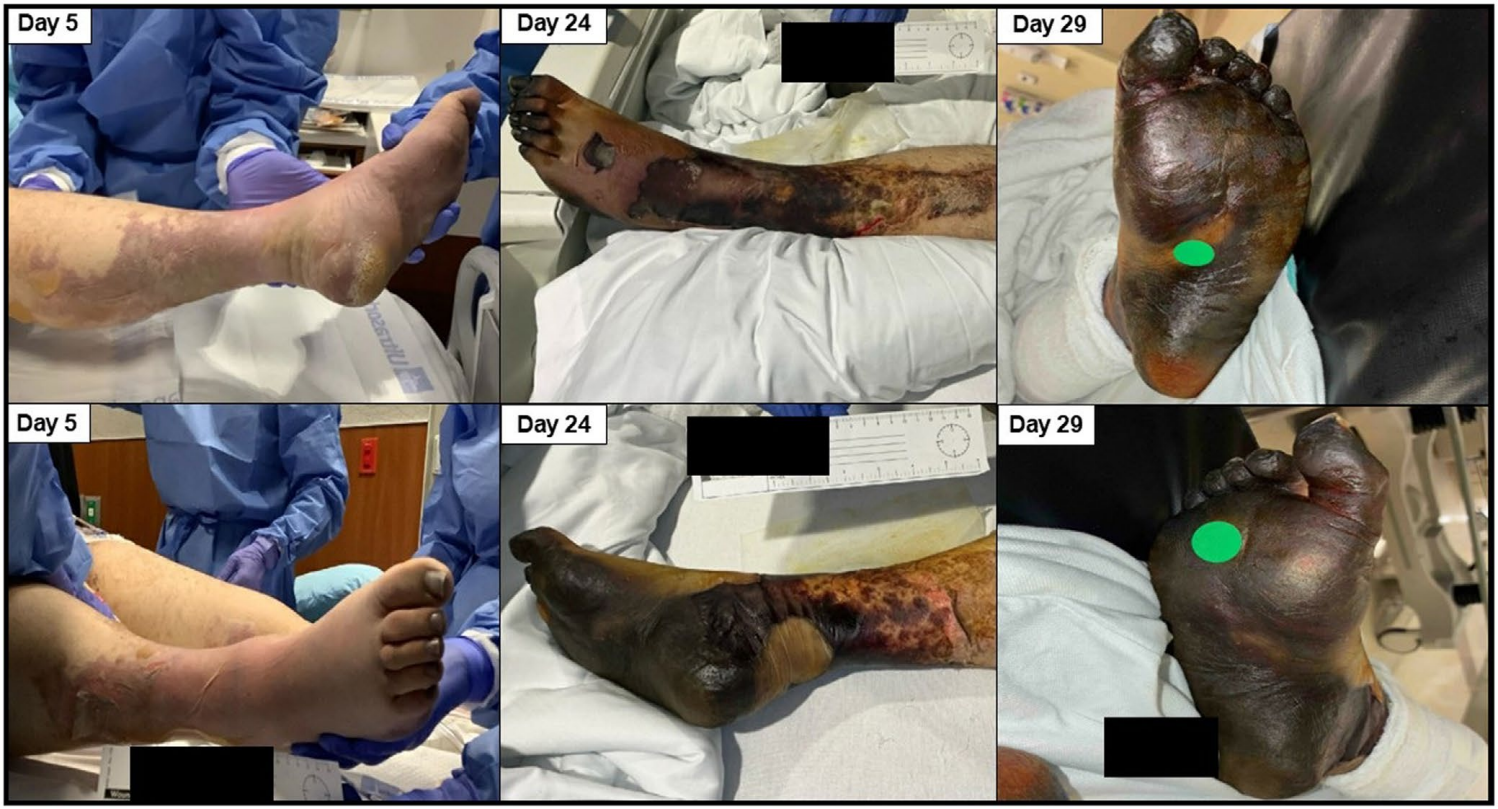
Progression of lower limb ischemia by hospital days.

During the hospital course, the patient developed acute respiratory distress syndrome (ARDS) by hospital day 4, necessitating continued mechanical ventilatory support and comprehensive critical care management. By hospital day 5, the clinical course was further complicated by vasopressor‐associated limb ischemia (VALI), reflecting the severity of distributive shock and sustained vasopressor requirements. Early signs of limb ischemia were identified through digital coolness and discoloration, prolonged capillary refill time, skin mottling, and peripheral perfusion index. Management of VALI included hyperbaric oxygen therapy, active external warming measures (e.g., Bair Hugger), optimization of hemodynamics with careful titration of vasopressors, systemic anticoagulation, limb elevation, and close vascular surgery monitoring. Despite these interventions, the ischemia progressed to irreversible gangrene. The patient subsequently required staged surgical amputations, including a left below‐knee amputation on hospital day 43, right below‐knee amputation on hospital day 57, left distal forearm amputation on hospital day 76, and right proximal phalangeal amputation on hospital day 83. Figures 1 and 2 show progression of upper and lower limb ischemia by hospital days.

This case highlights the profound morbidity associated with severe septic shock and multiorgan failure, despite timely, guideline‐directed critical care management. While aggressive resuscitation, antimicrobial escalation, and organ support achieved partial physiologic recovery, prolonged vasopressor dependence led to devastating ischemic complications. Early recognition of vasopressor‐associated limb ischemia and multidisciplinary management are essential; however, outcomes may remain poor, underscoring the need for preventive strategies and vigilant monitoring.

## 5 Discussion

This case illustrates the devastating consequences of vasopressor‐associated limb ischemia (VALI) in a critically ill patient with septic shock, ultimately resulting in four‐limb amputation despite aggressive multidisciplinary management. The convergence of morbid obesity, hyperinflammatory phenotype, and prolonged high‐dose vasopressor therapy created a perfect storm for catastrophic peripheral ischemia, highlighting the complex pathophysiology underlying this rare but life‐altering complication.

### 5.1 Pathophysiology of Vasopressor‐Associated Limb Ischemia

The pathogenesis of VALI represents a multifactorial process involving direct vasoconstrictive effects, microthrombotic phenomena, and underlying patient‐specific vulnerabilities [6, 7]. Vasopressors, particularly norepinephrine, phenylephrine, and vasopressin, exert their therapeutic effects through α‐adrenergic receptor activation and vasopressin receptor binding, resulting in systemic vasoconstriction to restore hemodynamic stability [2]. However, this same mechanism can lead to excessive peripheral vasoconstriction, particularly in acral regions where microvascular perfusion is already compromised by distributive shock [6]. The phenomenon of symmetrical peripheral gangrene (SPG), characterized by symmetrical distal ischemic damage without large vessel occlusion, represents the most severe manifestation of this complication [4, 6, 8].

The pathophysiology extends beyond simple vasoconstriction. Warkentin's seminal work on ischemic limb gangrene with pulses demonstrates that disseminated intravascular coagulation (DIC), natural anticoagulant failure, and microthrombosis play critical roles in the development of acral necrosis [6]. In critically ill patients with septic shock, systemic activation of hemostasis occurs through tissue factor expression on endothelium and monocytes, enhanced leukocyte‐endothelial interactions, and proinflammatory cytokine release [6]. This creates a prothrombotic milieu characterized by impaired fibrinolysis and intravascular fibrin deposition, predisposing to microvascular thrombotic occlusion [6]. The presence of shock liver, observed in approximately 90% of critically ill patients who develop acral ischemic necrosis, further contributes through impaired synthesis of protein C and antithrombin, critical natural anticoagulants [6]. Our patient's profound hepatic dysfunction (AST 238 U/L, ALT 129 U/L, total bilirubin 4.2 mg/dL) and consumptive coagulopathy (prolonged PT, hypofibrinogenemia, elevated D‐dimer) exemplify this pathophysiologic cascade.

### 5.2 Hyperinflammatory Phenotype and Cytokine Storm

The patient's laboratory profile revealed a striking hyperinflammatory phenotype characterized by extreme leukocytosis (57.11 × 10^3^/μL), markedly elevated procalcitonin (60.8 ng/mL), ferritin (1736 ng/mL), triglycerides (580 mg/dL), LDH (1315 U/L), and CRP > 200 mg/dL. This constellation of findings, meeting multiple HLH‐2004 diagnostic criteria, suggests a cytokine storm syndrome that likely amplified the risk of VALI through several mechanisms [9]. Cytokine‐mediated endothelial dysfunction, particularly through interleukin‐6 (IL‐6) trans‐signaling, results in increased vascular permeability, reduced E‐cadherin expression, and systemic hyperinflammation [9]. Tumor necrosis factor‐alpha (TNF‐α) and interleukin‐1 (IL‐1) further contribute to endothelial activation, upregulation of adhesion molecules, and enhanced leukocyte‐endothelial interactions [9, 10].

Recent evidence demonstrates that inflammatory cytokines, particularly IL‐1β, can induce neutrophil extracellular trap (NET) formation, which promotes endothelial dysfunction and aggravates limb ischemia through STAT1 signaling pathways [11]. Furthermore, critical limb ischemia is independently associated with elevated levels of multiple cytokines including IL‐1β, IL‐6, IL‐10, IFN‐γ, and VEGF, creating a circulating cytokine profile that resembles severe pancreatitis or sepsis [12]. This hyperinflammatory state likely synergized with vasopressor‐induced vasoconstriction to create a particularly hostile microvascular environment, predisposing to irreversible tissue ischemia. The systemic inflammatory response can produce uncontrolled vasodilation through cytokine‐driven excessive inducible nitric oxide synthase activation, paradoxically requiring higher vasopressor doses while simultaneously causing endothelial dysfunction and microvascular compromise [13].

### 5.3 The Obesity Paradox in Vasopressor Therapy

The patient's morbid obesity (BMI 40.82 kg/m^2^) introduces additional complexity to the pathophysiology of VALI. Emerging evidence suggests that obesity fundamentally alters vasopressor pharmacodynamics and hemodynamic responses in septic shock [14, 15, 16, 17]. Obese patients require lower weight‐based doses of norepinephrine to achieve similar hemodynamic endpoints compared to nonobese patients, yet they paradoxically receive similar or higher total doses when non‐weight‐based dosing strategies are employed [17, 18]. This creates a potential for relative under‐dosing in obese patients when fixed‐dose strategies are used, leading to prolonged vasopressor requirements and delayed hemodynamic stability [15].

Wacharasint et al. [16] demonstrated that obese septic shock patients have a muted inflammatory IL‐6 response and receive less fluid and vasopressor per kilogram compared to nonobese patients yet paradoxically have improved survival. However, recent data suggest that obesity is associated with an attenuated hemodynamic response to adjunct vasopressin, with obese patients showing increased norepinephrine requirements after vasopressin initiation rather than the expected reduction observed in normal‐weight patients [14]. This obesity‐related resistance to vasopressin may be driven by intrinsic physiological factors rather than insufficient dosing [14]. In our patient, the combination of morbid obesity and prolonged vasopressor therapy (12 days of norepinephrine, phenylephrine, and vasopressin in varying combinations) likely contributed to the development of VALI through altered pharmacodynamics, prolonged exposure, and potentially inadequate weight‐based dosing strategies.

### 5.4 Risk Factors and Medication‐Specific Considerations

Large‐scale studies have identified medication‐specific factors as the primary determinants of VALI rather than patient demographics or comorbidities [3]. Derry et al. [3] found that neither baseline demographics nor medical comorbidities differed significantly between patients who developed limb necrosis and those who did not, with an overall incidence of 0.25% among patients receiving vasopressor therapy. However, higher peak doses and prolonged duration of vasopressor therapy, particularly with norepinephrine, epinephrine, and dopamine, significantly increased the risk of limb necrosis [3]. In a matched case–control study, Kwon et al. [4] demonstrated that weight‐compensated peak doses of norepinephrine, dopamine, and epinephrine significantly contributed to SPG development, with up to 70% of survivors requiring amputation.

Vasopressin therapy carries particular risks, with ischemic skin lesions developing in approximately 30% of patients receiving continuous vasopressin infusion for catecholamine‐resistant vasodilatory shock [19]. Independent risk factors for ischemic skin lesions during vasopressin therapy include preexistent peripheral arterial occlusive disease and the presence of septic shock [19]. Our patient received multiple vasopressors simultaneously for 12 days, representing a particularly high‐risk scenario. The American Heart Association scientific statement on prevention of complications in the cardiac intensive care unit emphasizes that careful titration of vasoactive medications with assessment of tissue perfusion effects can allow use of minimal effective dosages to minimize adverse effects including limb ischemia and necrosis [2].

The selection of phenylephrine in this case needs additional discussion. Phenylephrine is a selective alpha 1 adrenergic agonist and is not routinely used as a first‐line adjunctive vasopressor in septic shock. Current guidelines recommend norepinephrine as the first‐line therapy, followed by vasopressin. However, in our patient, phenylephrine was administered as rescue therapy for refractory distributive shock since the patient developed marked tachycardia and had preserved left ventricular systolic function. Phenylephrine lacks beta 1 adrenergic activity and hence is preferred for increasing vascular tone without chronotropic effects. This scenario underscores the importance of individualized vasopressor selection guided by the patient's hemodynamic phenotype, cardiac function, adverse effect susceptibility, and evolving response to therapy [20].

### 5.5 Clinical Implications and Prevention Strategies

The catastrophic outcome in this case underscores the critical importance of early recognition and prevention of VALI. Current guidelines recommend norepinephrine as the first‐line vasopressor in septic shock, with careful monitoring for efficacy and titration to the lowest effective dose [1, 21, 22]. The Surviving Sepsis Campaign guidelines acknowledge that vasopressin combined with norepinephrine may reduce mortality compared to norepinephrine alone, though an individual patient data meta‐analysis demonstrated a higher risk of digital ischemia with vasopressin therapy (risk difference 1.7%) [1]. This highlights the delicate balance between hemodynamic support and peripheral perfusion.

Prevention strategies should focus on several key principles. First, vasopressor therapy should be individualized based on clinical evaluation and blood flow measurements to avoid excessive vasoconstriction [21]. Second, in obese patients, consideration should be given to weight‐based dosing strategies to avoid prolonged exposure and delayed hemodynamic stability [15, 18]. Third, early recognition of hyperinflammatory phenotypes may identify patients at particularly high risk for microvascular complications, potentially warranting more aggressive monitoring and earlier consideration of adjunctive therapies such as corticosteroids or targeted anti‐cytokine therapy [9]. Fourth, meticulous attention to tissue perfusion markers including skin mottling, capillary refill time, and lactate clearance can help identify early signs of excessive vasoconstriction before irreversible ischemia develops [21].

Management of established VALI remains challenging, with limited evidence supporting specific interventions. Our patient received hyperbaric oxygen therapy, active external warming, optimization of hemodynamics, systemic anticoagulation, and limb elevation, yet ischemia progressed to irreversible gangrene requiring staged amputations. This reflects the reality that once established, VALI often progresses despite maximal medical therapy. The high mortality rate and devastating functional outcomes associated with VALI emphasize that prevention should be achieved through vigilant monitoring methods such as digital coolness and discoloration, prolonged capillary refill time, skin mottling, core‐to‐peripheral temperature gradient, transcutaneous O_2_/CO_2_ tension, sublingual microcirculation, near‐infrared spectroscopy, and peripheral perfusion index [3, 4, 8]. Additionally, vasopressors should be used cautiously.

### 5.6 Limitations and Future Directions

This case highlights several important knowledge gaps in the management of VALI. The optimal vasopressor selection and escalation strategy in obese patients with hyperinflammatory phenotypes remains unclear. Whether targeted anti‐inflammatory therapies, such as IL‐6 inhibitors (tocilizumab) or IL‐1 antagonists, could mitigate the risk of VALI in patients with cytokine storm syndromes warrants investigation. Additionally, the role of predictive biomarkers, such as serum renin or specific cytokine profiles, in guiding vasopressor selection and identifying high‐risk patients requires further study [21]. Finally, the development of novel vasopressors with more selective vascular effects or the use of mechanical circulatory support to reduce vasopressor requirements in refractory shock may offer future strategies to prevent this devastating complication.

## 6 Conclusion

This case of four‐limb amputation following vasopressor therapy in a morbidly obese patient with hyperinflammatory phenotype illustrates the complex interplay between vasopressor pharmacology, obesity‐related pharmacodynamic alterations, and cytokine‐mediated microvascular injury. The convergence of prolonged high‐dose vasopressor therapy, morbid obesity, and severe systemic inflammation created a particularly high‐risk scenario for catastrophic VALI. While vasopressors remain essential for hemodynamic support in septic shock, clinicians must maintain vigilant awareness of this rare but devastating complication, with emphasis on early recognition, judicious dosing, individualized therapy based on patient characteristics, and meticulous monitoring of tissue perfusion. Prevention through careful vasopressor titration and early identification of high‐risk patients represents the most effective strategy, as established VALI often progresses despite maximal medical intervention.

## Author Contributions

**Muni Rubens:** conceptualization, methodology, writing – original draft. **Venkataraghavan Ramamoorthy:** writing – original draft, methodology, software. **Krimal Shah:** validation, resources, writing – review and editing. **Ashley Rodriguez:** writing – review and editing, investigation, data curation. **Brian Murillo:** software, data curation, writing – review and editing, validation. **Karelis Lopez Muniz:** validation, visualization, data curation, investigation. **Anshul Saxena:** methodology, writing – review and editing, data curation. **Peter McGranaghan:** funding acquisition, investigation, data curation, validation. **Tina Sanjar:** validation, supervision, writing – review and editing.

## Funding

The authors have nothing to report.

## Ethics Statement

This study was conducted in accordance with the principles of the Declaration of Helsinki. Institutional review board (IRB) approval was not required for this single‐patient case report in accordance with institutional policy.

## Consent

Written informed consent was obtained from the patient to publish this report, following the journal's patient consent policy.

## Conflicts of Interest

The authors declare no conflicts of interest.

## Data Availability

Data sharing is not applicable to this article as no datasets were generated or analyzed during this study.
